# Thermal and Flame Retardant Behavior of Neem and Banyan Fibers When Reinforced with a Bran Particulate Epoxy Hybrid Composite

**DOI:** 10.3390/polym13223859

**Published:** 2021-11-09

**Authors:** Thandavamoorthy Raja, Vinayagam Mohanavel, Thanikodi Sathish, Sinouvassane Djearamane, Palanivel Velmurugan, Alagar Karthick, Omaima Nasif, Saleh Alfarraj, Ling Shing Wong, Shanmugam Sureshkumar, Manikkam Ravichandran

**Affiliations:** 1 Department of Mechanical Engineering, Vel Tech Rangarajan Dr. Sagunthala R&D Institute of Science and Technology, Chennai 600062, India; 2 Centre for Materials Engineering and Regenerative Medicine, Bharath Institute of Higher Education and Research, Chennai 600073, India; mohanavel2k16@gmail.com (V.M.); palanivelvelmurugan@bharathuniv.ac.in (P.V.); 3 Department of Mechanical Engineering, Saveetha School of Engineering, SIMATS, Chennai 602105, India; sathish.sailer@gmail.com; 4 Faculty of Science, Universiti Tunku Abdul Rahman, Kampar 31900, Malaysia; 5 Department of Electrical and Electronics Engineering, KPR Institute of Engineering and Technology, Coimbatore 641407, India; karthick.power@gmail.com; 6 Department of Physiology, College of Medicine and King Khalid University Hospital, King Saud University, Medical City, Riyadh 11461, Saudi Arabia; onasif@ksu.edu.sa; 7 Zoology Department, College of Science, King Saud University, Riyadh 11451, Saudi Arabia; salfarraj@hotmail.com; 8 Faculty of Health and Life Sciences, INTI International University, Nilai 71800, Malaysia; shing.wong@newinti.edu.my; 9 Department of Animal Resources Science, Dankook University, 119 Dandae-ro, Cheonan 31116, Korea; suresh09@dankook.ac.kr; 10 Department of Mechanical Engineering, K. Ramakrishnan College of Engineering, Trichy 621112, India; smravichandran@hotmail.com

**Keywords:** thermal analysis, short-term heat resistance, hybrid composite, natural fibers

## Abstract

Awareness of environmental concerns influences researchers to develop an alternative method of developing natural fiber composite materials, to reduce the consumption of synthetic fibers. This research attempted testing the neem (*Azadirachta indica*) fiber and the banyan (*Ficus benghalensis*) fiber at different weight fractions, under flame retardant and thermal testing, in the interest of manufacturing efficient products and parts in real-time applications. The hybrid composite consists of 25% fiber reinforcement, 70% matrix material, and 5% bran filler. Their thermal properties—short-term heat deflection, temperature, thermal conductivity, and thermal expansion—were used to quantify the effect of potential epoxy composites. Although natural composite materials are widely utilized, their uses are limited since many of them are combustible. As a result, there has been a lot of focus on making them flame resistant. The thermal analysis revealed the sample B was given 26% more short-term heat resistance when the presence of banyan fiber loading is maximum. The maximum heat deflection temperature occurred in sample A (104.5 °C) and sample B (99.2 °C), which shows a 36% greater thermal expansion compared with chopped neem fiber loading. In sample F, an increased chopped neem fiber weight fraction gave a 40% higher thermal conductivity, when compared to increasing the bidirectional banyan mat of this hybrid composite. The maximum flame retardant capacity occurred in samples A and B, with endurance up to 12.9 and 11.8 min during the flame test of the hybrid composites.

## 1. Introduction

Growing demands for environmental awareness in the recent era, promoting the use of renewable resources in numerous applications of lightweight materials, have gained the attention of researchers in the industry. Natural fibers are considered to be an alternative in the current material research, as they are potentially beneficial to selected structural applications in automotive and other material processing industries [1]. High-temperature execution is especially extraordinary for carbon fibers [2]. Polyester and nylon thermoplastic fibers have been recently presented as essential fortifications; additionally, in a hybrid procedure with fiberglass and an increasingly particular combination of high-quality and high-temperature uses, incorporating metals and metal oxides, they can be utilized for applications such as those in aviation fields [3]. The most widely recognized thermoset polymer that is utilized for natural fiber reinforcement is unsaturated polyester (isophthalic or orthothalic), which is used because of its low consistency, quick restoring time, and minimal required effort [4]. Natural fiber-reinforced composites have been profoundly examined in the most recent decade because of their particular properties and their positive natural effects. The heat conduction of fiber-strengthened composites was investigated in this study. The expanding conduct has been examined in various arrangements, having a scope of pH values. The ideal outcomes were acquired, with 35 wt % filler contents for UP/Jute/ZrO_2_ composites [5]. In the creation and examination of the mechanical and thermal properties of an epoxy composite reinforced with banana–kenaf glass fiber, warm tests were carried out and the outcome showed that the hybrid composite fibers were orchestrated at a 45° tendency and have preferred properties over the others [6]. In a review study on the thermal behavior of natural fibers filled with materials such as cellulose and protein particles, eco-friendly flame retardant treatments are shown to overcome the impact of those environmental issues can influence the performance of natural fiber composite [7]. The volume portions of the Pennisetum purpureum, glass fiber, and epoxy resin were 24, 6, and 70%, respectively. Thermo Gravimetric Analysis (TGA) showed that the measure of a build-up of the hybrid composites diminished as the grouping of the alkali (used to treat the Pennisetum purpureum filaments) expanded [8]. When tried at room temperature (RT), the greatest malleable and flexural qualities were recorded for hybrid composites with 5% soluble base treated Pennisetum purpureum filaments. At >60 °C, as the temperature drew nearer Tg, the fiber and network de-bonded, which brought about a decrease of the tractable and flexural qualities [8,9]. Fourier change infrared spectroscopy (FT-IR) examination indicated an increment in intermolecular hydrogen holding, following the expansion of SPF. The thermal steadiness of the hybrid composites was improved, as shown by a higher beginning corruption temperature (259 °C) for 25:75 seaweed–SPF composites than for the individual kelp composites (253 °C) [10]. The hydrophilic property of regular fiber gives a poor grip between the fiber matrices, natural fibers have more areas of flexibility when contrasted with manmade fibers (e.g., glass, carbon, and Kevlar fiber) [11]. Cement paste, reinforced with cellulose nanocrystal particles, was examined for fracture behavior, compressive strength, and hydration characteristics. The addition of CNCs resulted in a longer inactive phase, which caused the hydration reaction to be delayed. In comparison to the reference sample, the addition of CNCs resulted in a greater heat flow [12]. Polymeric materials made from fossil fuels or sustainable resources are no longer imaginable in modern civilization, including applications in domains where flame retardancy is required. A significant problem for materials research and development is that the creation of flame retardants and flame retardant polymeric materials must comply with fire safety laws and pass rigorous fire testing. Epoxy resins and other high-performance polymers, such as halogen-free and self-extinguishing thermoplastics, thermosets, fiber-reinforced composites, fibers, and foams, are now being developed [13].

In this present study, the chopped neem fiber and bidirectional woven fibers were used as reinforcement and bran is used as a filler, blended with an epoxy matrix to fabricate a composite laminate through a traditional hand layup technique. This is followed with varying the weight fraction in order to calculate the thermal properties of the heat deflection temperature, the coefficient of linear thermal expansion, the thermal conductivity, and to analyze the flame retardant capacity of the hybrid composite. Based on the results, new materials can be enhanced for thermal insulation applications.

## 2. Materials and Methods

### 2.1. Materials Overview

**Figure 1:**
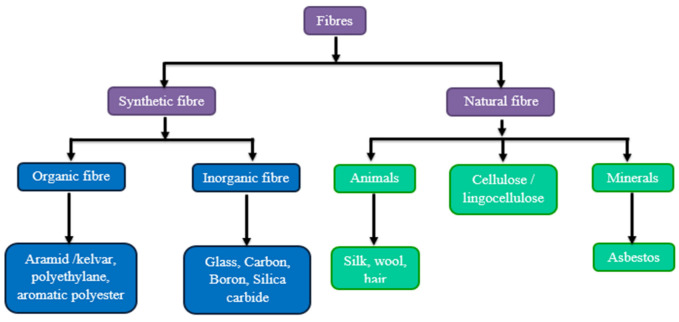
Flow chart of different types of fibers for the reinforcement phase.

**Figure 2:**
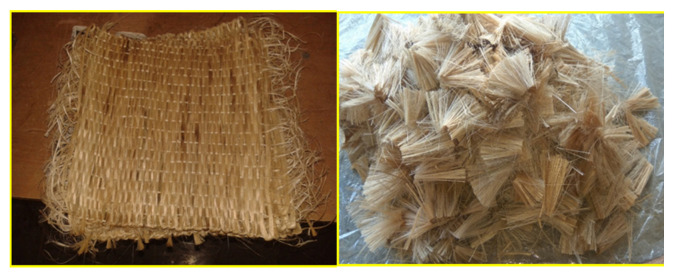
Banyan woven fabric and chopped neem fiber for reinforcement.

The two types of fiber materials used in this study are easily available and biodegradable. Different types of fibers that can be used for the reinforcement phase of the composite are given in Figure 1. For the experiment, the neem fiber was naturally processed and chopped into uniform size, and the banyan fiber was collected from Go Green Pvt. Ltd., Chennai, India. Banyan fiber was prepared by the conventional drying process and was woven into bidirectional woven fabric. Bran fillers can improve mechanical properties, including fire and smoke performance, by diminishing the natural substances in composite concealments [14]. Epoxy L556 is a pale yellow, clear liquid. The viscosity of this fluid is 10,000–12,000 mPa.s. The density of the liquid is 1.15–1.2 g/cm^3^ [15]. High-performance composite parts can be fabricated with epoxy LY 556 as base material, with hardener HY951 used as a catalyst [16] for bonding purposes (collected from Javanthi enterprises, Chennai, India). The bidirectional woven fabric and neem chopped fiber are shown in Figure 2.

### 2.2. Experimental Method

**Figure 3:**
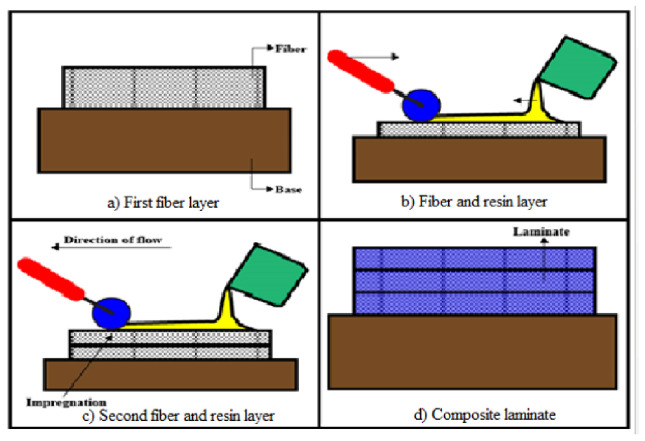
The schematic fabrication process of the hybrid composite.

**Figure 4:**
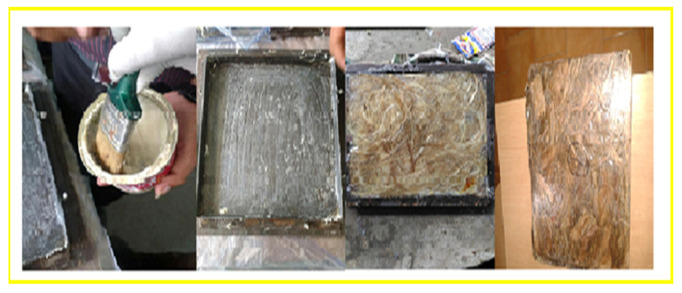
The fabrication process of the hybrid epoxy composite.

The research initiated with the collection of natural fibers, such as neem and banyan fiber, which are available locally. The fibers were prepared using fabric and were chopped. Sequential to the fiber preparation, it was chemically treated with an alkaline solution to improve the properties of the reinforcement [17]. The schematic diagram of the hand layup fabrication technique is shown in Figure 3. The fabrication of the composites was performed with the hand layup method as follows: the fibers of neem were layered, the bran fillers with epoxy resin were applied on the fiber top layer, and the banyan fiber was placed on the top via the lamination process. A stacking sequence of seven fiber samples was prepared with different fiber weight fractions. The process of fabrication is shown in Figure 4. A resin particle that combined Bisphenol-F LY556 Epoxy polymer and Araldite HY 951 hardener was used to achieve better bonding properties of the natural fibers [18]. The weight concentrations of the neem and banyan epoxy composites are given in Table 1.

The motive for testing these samples was to verify which weight fraction of the hybrid composition obtained a better result in terms of thermal behavior. The thermal analyses including heat deflection temperature, coefficient of linear thermal expansion, thermal conductivity, and flame retardant capacity were performed according to the following ASTM standards: ASTM D1525, ASTM E1530, ASTM D696, and ASTM E119. These were conducted with an Atlas HMV horizontal flammability tester, to evaluate whether the tested specimens were capable of thermal behavior at high thermal loads. A heat flow meter (Unitherm model 2022, ANTER Corp., Pittsburgh, PA, USA) was used to test the thermal conductivity of the hybrid composite at a mean temperature of 55 °C, according to ASTM E1530 and the standard test method for evaluating resistance to thermal transmission by the guarded heat flow meter technique, which is standard for assessing a hybrid composite’s thermal insulation [19]. The VICAT softening apparatus was used to evaluate the heat deflection temperature of the hybrid composite, according to the ASTM D1525. This was used to examine the hybrid composite’s ability to withstand low loads at a high temperature and to determine the hybrid composite’s short-term heat resistance. The samples of the hybrid composite were subjected to the normal stress of 1.82 MPa, after which they were immersed in silicon oil to raise the temperature at a rate of 5 °C/min until the hybrid composite specimen deformed to 0.25 mm [20]. The ASTM D696—the standard test method for the coefficient of linear thermal expansion of plastics between −30 °C and 30 °C using a vitreous silica dilatometer—was used to determine the coefficient of linear thermal expansion of the hybrid composite [21].

## 3. Result and Discussion

### 3.1. Surface Morphology of a Hybrid Composite

**Figure 5:**
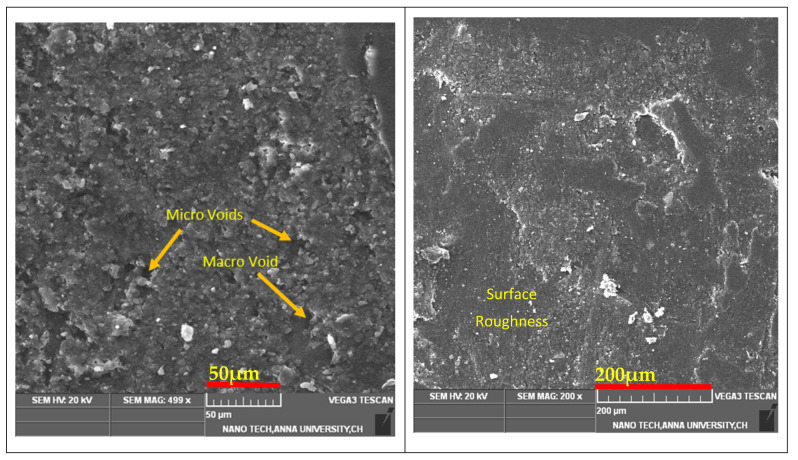
SEM image of hybrid composite laminates.

The SEM micrograph revealed the fracture mode of the hybrid composite. In this study, SEM micrographs were taken for surface analysis to identify the cracks or voids during the fabrication process, surface smoothness, and bonding between the fibers and matrix with the bran filler. Therefore, before conducting the tests to identify the surface finishing, this morphological analysis was required to reveal the bonding capacity of the hybrid composite. During the fabrication process, there are few ways to acquire the voids due to the atmospheric condition; therefore, after completing the laminates to conduct the SEM analysis, it was useful to observe all the behaviors of the hybrid composite. The SEM micrographs of the hybrid composite laminates are shown in Figure 5. In this analysis, one could clearly see the natural fibers (neem and banyan) was developed good bonding with epoxy resin and bran fillers. Therefore, the analysis can be suitable for conducting the properties of mechanical, thermal, and dynamical behaviors of the hybrid composite.

### 3.2. Heat Deflection Temperature of an Epoxy Composite

**Figure 6:**
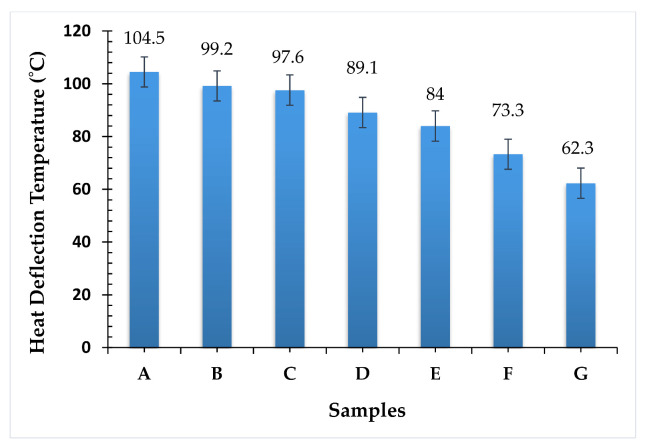
Heat deflection temperature of hybrid epoxy composite.

The heat deflection graph revealed that the banyan fibers can withstand high temperatures, compared with the broken neem fibers. As such, the composite that had all four layers of banyan fibers exhibited the maximum heat deflection temperature, compared with the other composites considered in this study. The measured heat deflection temperature for composite A was 104.5 °C. The addition of discontinuous neem fibers as the reinforcement proved to be detrimental to the heat deflection ability of the composite material [18]. The extent to which the heat deflection temperature was affected depended on the number of discontinuous neem fibers in the composite material. Interestingly, the stacking sequence played a vital role in determining the heat deflection temperature exhibited by the respective composites [19]. There was negligible variation in the cases of composites B and C: even though the latter had two layers of discontinuous neem fibers, they were separated by two intermediate layers of banyan fibers. Figure 6 shows the heat deflection temperature of the hybrid epoxy composite.

There was a dramatic change in the heat deflection temperature in composite D that had only one layer of banyan fibers separating the discontinuous neem fibers. As a result of the heat deflection, the temperature reduced by 14.74%, compared with composite A. In composite E, banyan fibers were sandwiching two interlayers of discontinuous neem fibers. This facilitated sufficient heat transfer but was restricted by the intermediate layers of discontinuous neem fibers; hence, there was a reduction of 19.62% compared with composite A. However, composite G, which had all four layers composed of discontinuous neem fibers, exhibits the least heat deflection temperature. This composite had a 40.4% lower heat deflection temperature, compared with composite A.

### 3.3. Co-Efficient of Linear Thermal Expansion of Hybrid Epoxy Composite

**Figure 7:**
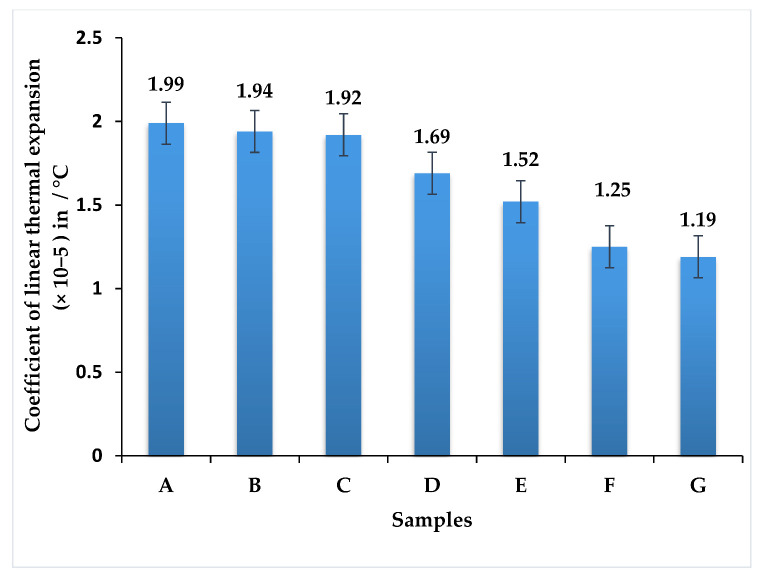
The thermal expansion coefficient of hybrid epoxy composite.

The thermal expansion coefficient is presented in Figure 7, to exhibit identical trends, compared with the heat deflection temperature of the composite materials considered in this research. As such, the composite that had all four layers composed of banyan fibers exhibited the maximum thermal expansion coefficient, compared with the other composites considered in this study. The measured thermal expansion coefficient for composite A was 1.99 × 10^−5^. The addition of discontinuous neem fibers as the reinforcement proved to be detrimental to the heat deflection ability of the composite material. From the findings, sample F possessed a lower thermal expansion coefficient of 1.25 × 10^−5^, for the neem and banyan fiber weight fraction of 2:1, and sample A had a maximum value of hybrid composite. With a ratio of 2:1 weight fraction, a hybrid composite can withstand high temperature while attaining low thermal expansion. An increase of the weight fraction of neem gave a positive impact on the fabrication of hybrid composite, and the increase of banyan fiber gave a negative impact. The outcome showed that the hybrid composites of samples F and G provided better thermal resistance and can be used as a better thermal insulator.

The extent to which the thermal expansion coefficient gets affected depends on the number of discontinuous neem fibers in the composite material [19,20]. In the case of composite B, having just one layer of discontinuous neem fibers reduced the thermal expansion coefficient by 2.5%. Interestingly, the stacking sequence played a vital role in determining the heat deflection temperature exhibited by the respective composites. There was negligible variation in the cases of composites B and C (although the latter had two layers of discontinuous neem fibers, they were separated).

There was a dramatic change in the thermal expansion coefficient in composite D, which had only one layer of banyan fibers separating the discontinuous neem fibers. As a result of the thermal expansion, the coefficient was reduced by 15%, compared with composite A. In composite E, banyan fibers were sandwiching two interlayers of discontinuous neem fibers. This facilitated sufficient heat transfer but was restricted by the intermediate layers of discontinuous neem fibers [20]; hence, there was a reduction of 23.62% compared with composite A. However, composite G, which had all four layers of discontinuous neem fibers, exhibits the lowest thermal expansion coefficient. This composite had a 40.2% lower thermal expansion coefficient compared with composite A.

### 3.4. Thermal Conductivity of the Hybrid Epoxy Composite

**Figure 8:**
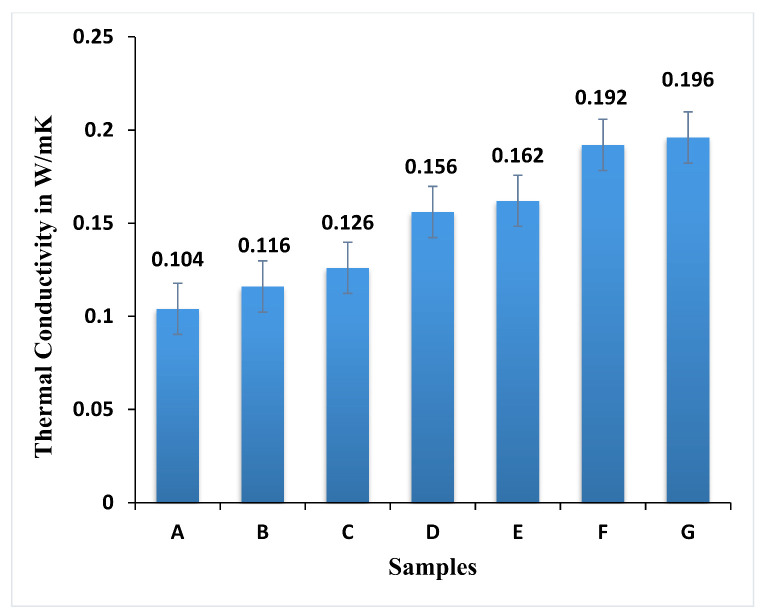
Thermal conductivity of the hybrid epoxy composite.

The thermal conductivity test was employed to investigate the hybrid composite, as shown in Figure 8, that has endured a maximum range of conductivity at high heat temperature. The test was conducted based on the ASTM standard for seven sorts of specimen weight fraction at 55 °C was calculated, and the result provides less thermal conductivity [21]. When comparing the performance of thermal conductivity of all seven specimens, specimens G and F obtained better thermal conductivity of 0.192 and 0.196 W/mK for the 2:1 ratio of weight fraction of neem fiber by 90 g with 45 g of banyan woven fabric respectively. In the meantime, the weight fraction was reversed to 90 g of banyan, and 45 g of neem attains a low thermal conductivity in the range of 0.116 W/mK. The concluded results show that samples A and B possess better thermal resistance properties in the hybrid composites, respectively.

### 3.5. Flame Retardant Capacity of Hybrid Composites

**Figure 9:**
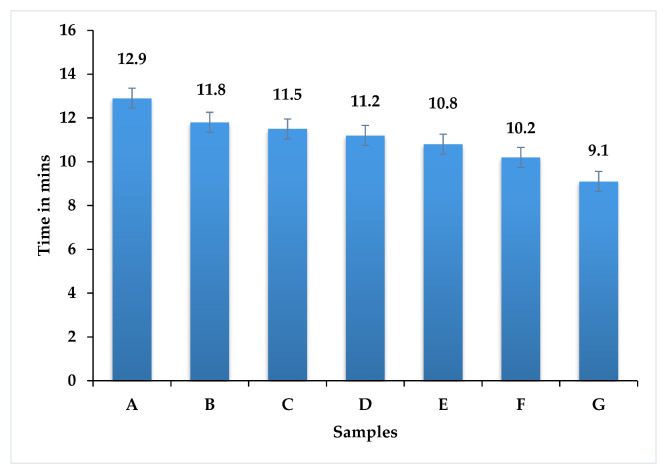
Flame retardant effect of hybrid epoxy composite.

The developed hybrid composites were examined for the capacity of flame retardancy exposed to high temperature, this reaction reveals chemical decomposition, and the results lead to combustion with the effect of oxygen [22]. The effect of composite decomposing time taken during the flame retardancy experiment is shown in Figure 9.

In this research, the composite laminate contained natural fibers, bran filler, and an epoxy matrix. The effects were revealed with the flame test. All seven samples had good resistance with temperature, due to the presence of micro bran particles used as filler material for the composite laminates, which was constant for all samples; additionally, the graph reveals a small time difference between the samples reaching the gas phase [23]. When micro or nanoparticles are used as filler, the content required to fabricate a composite material gives more flame resistance compared with no filler loading of composite materials [24]. At the same time, in samples A, B, and C (containing the maximum amount of woven banyan fiber), the times taken to reach the decomposition phase were 12.9 min, 11.8 min, and 11.5 min respectively. Therefore, increasing the woven banyan fiber has a positive influence on flame resistance, compared with increasing chopped neem fiber loading.

## 4. Conclusions

The thermal behaviors (heat deflection temperature, the coefficient of linear thermal expansion, thermal conductivity, and the flame retardant capacity) of neem and banyan fiber composites, reinforced with bran filler loading hybrid epoxy, were studied. The following major findings are observed:

Short-term heat resistance was greater in sample A and sample B, according to the measured values of heat deflection temperatures (104.5 °C and 99.2 °C, respectively). The number of discontinuous neem fibers in the composite material determines how much the heat deflection temperature was altered, which indicates that the woven banyan fiber can withstand 39% and 26% more loading temperature (compared with chopped neem fiber) and distribute evenly from one point to another point of the hybrid composite.

Thermal expansion capacity was low in all samples. Composite A has a measured thermal expansion coefficient of 1.99 × 10^−5^/°C. The use of discontinuous neem fibers as a reinforcement proved to be harmful to the composite material’s heat deflection capabilities. It showed that the volume did not change due to the effect of temperature and that the composite contains a more solid glassy region and a less rubbery region of the hybrid composite.

Samples F and G have increased thermal conductivity of 47% and 40%, respectively, compared with samples A and B. When the weight fractions were inverted to 90 g banyan and 45 g neem, the thermal conductivity drops to 0.116 W/mK. This indicates that the chopped neem fiber bonding had more adhesion between the fiber and the matrix, and that it transferred more heat from one point to another point.

In terms of flame retardancy, all samples had an equal amount of bran micro filler material, which resists decomposition of the hybrid composite during flame retardant analysis. Samples A and B were 29% and 14% more effective compared with samples G and F, respectively. In comparison to increasing chopped neem fiber loading, increasing woven banyan fiber has a positive effect on flame resistance.

The future scope of this work, the treatment of the fibers before fabrication can improve their thermal stability when conducting thermogravimetric analysis for analyzing the thermal stability of the hybrid composite.

## Figures and Tables

**Table 1:** The weight concentration of the hybrid epoxy composite.

Sample	Filler in Grams	Epoxy Matrix Weight in Grams	Banyan Fiber in Grams	Neem Fiber in Grams	Weight Fraction of Banyan/Neem Fibers in %	Composite Laminate Weight in Grams
A	25	385	135	0	25/0	545
B	25	385	90	45	17/8	545
C	25	385	75	60	14/11	545
D	25	385	67.5	67.5	12.5/12.5	545
E	25	385	60	75	11/14	545
F	25	385	45	90	8/17	545
G	25	385	0	135	0/25	545

## Data Availability

Data sharing is not applicable for this article.
